# Superselective Unilateral Embolization of the Sphenopalatine Artery for Severe Posterior Epistaxis: A Prospective Study on the Safety and Efficacy

**DOI:** 10.3390/jcm14144864

**Published:** 2025-07-09

**Authors:** Antonio Vizzuso, Maria Vittoria Bazzocchi, Antonio Spina, Giorgia Musacchia, Andrea De Vito, Giuseppe Meccariello, Enrico Petrella, Emanuela Giampalma, Matteo Renzulli

**Affiliations:** 1 Radiology Unit, Morgagni-Pierantoni Hospital, AUSL Romagna, 47121 Forlì, Italy; mariavittoria.bazzocchi@auslromagna.it (M.V.B.); antonio.spina@auslromagna.it (A.S.); giorgia.musacchia@auslromagna.it (G.M.); enrico.petrella@auslromagna.it (E.P.); emanuela.giampalma@auslromagna.it (E.G.); matteo.renzulli@auslromagna.it (M.R.); 2 Head-Neck and Oral Surgery Unit, Department of Head-Neck Surgery, Otolaryngology, Morgagni-Pierantoni Hospital, 47121 Forlì, Italy; andrea.devito@auslromagna.it (A.D.V.); giuseppe.meccariello@auslromagna.it (G.M.); 3 Department of Medical and Surgical Sciences, University of Bologna, 40100 Bologna, Italy

**Keywords:** epistaxis, superselective embolization, interventional radiology, polyvinyl alcohol particles, fluoroscopy, hemostasis

## Abstract

**Objectives:** Epistaxis is a common condition affecting up to 60% of the population, with approximately 6% requiring medical intervention. Posterior epistaxis is particularly challenging, often necessitating endoscopic or endovascular treatment. Sphenopalatine artery (SPA) embolization is an effective treatment option, though concerns remain about the risks associated with nonselective or bilateral approaches. This study evaluates the efficacy and safety of unilateral superselective SPA embolization in managing severe posterior epistaxis. **Methods:** A prospective study of patients undergoing unilateral superselective SPA embolization for refractory posterior epistaxis over a four-year period was conducted. Demographic data, clinical history, prior treatments, and procedural characteristics were analyzed. The primary endpoint was clinical success, defined as the absence of recurrent bleeding within 24 h post-procedure. Secondary outcomes included recurrence at one month and complication rates. **Results:** Thirty-two patients with severe posterior epistaxis were included. All required nasal packing prior to embolization. Half had undergone previous endoscopic cauterization. Hypertension was present in 69%, and 56% were receiving anticoagulant or antiplatelet therapy. Clinical success was achieved in 100% of cases, with no rebleeding in the first 24 h. Two patients (6%) experienced early recurrence within seven days, requiring readmission. Minor complications included nasal dryness in two cases (6%); no major complications occurred. Mean fluoroscopy time was 19.9 ± 11 min. **Conclusions:** Unilateral superselective SPA embolization is a safe and highly effective treatment for severe posterior epistaxis, offering high initial success and low complication rates. Its adoption may reduce the need for bilateral procedures and surgical interventions.

## 1. Introduction

Epistaxis, defined as bleeding from the nasal cavity, affects up to 60% of the general population, with medical care being required in roughly 6% of cases [1].

While most cases are self-limiting, severe or recurrent epistaxis require specialized management due to the risks of hemodynamic instability, anemia, and airway compromise [1,2]. Given the vascular complexity of the nasal cavity, a thorough understanding of its etiology and appropriate treatment strategies is crucial for achieving effective hemostasis [3,4].

Epistaxis can be classified as anterior or posterior based on its site of origin. Approximately 80–90% of cases originate from anterior sources, primarily Kiesselbach’s plexus, and can typically be managed conservatively with local pressure, topical vasoconstrictors, and nasal packing. However, posterior epistaxis accounts for 5–10% of cases and is often more severe, arising from branches of the internal maxillary artery (IMA), in particular from the sphenopalatine artery (SPA) as well as the facial artery (FA) and other branches of the carotid system. This type of bleeding is usually refractory to conservative measures and necessitates more aggressive interventions, including endoscopic cauterization, surgical ligation, or arterial embolization (ArtE) [1,2].

Nasal packing, although historically considered the first-line treatment, has notable limitations, including prolonged hospital stays, significant patient discomfort, risk of aspiration, and, in some cases, failure to control hemorrhage [1]. Endoscopic approaches, particularly bipolar or monopolar coagulation of the SPA, have gained prominence as a primary treatment strategy due to their minimally invasive nature and the ability to provide the direct visualization of the bleeding source [5,6]. However, despite advancements in endoscopic techniques, a subset of patients continues to experience persistent or recurrent bleeding, necessitating an alternative therapeutic approach [1,2].

Interventional radiology is emerging as a highly effective technique in various fields of medicine [7]. In particular, ArtE has emerged as a highly effective intervention for severe posterior epistaxis, particularly in patients with failed endoscopic management, coagulopathies, or contraindications to surgery [8,9]. Historically, concerns regarding ArtE have included potential complications such as tissue necrosis, stroke, inadvertent embolization of non-target vessels, and bilateral occlusion leading to significant mucosal ischemia [9,10,11]. However, advances in catheterization techniques, microcatheter technology, and improved imaging modalities have significantly enhanced the safety profile of this procedure [7,12].

Unfortunately, despite these advancements, certain limitations persist. The risk of ischemic complications following embolization, though reduced, remains, particularly when ArtE involves multiple branches of the IMA. Notably, bilateral ArtE has been associated with a higher incidence of mucosal necrosis and sensory disturbances due to the compromised vascular supply [10,11].

This study aims to evaluate the efficacy and safety of the unilateral superselective ArtE of the SPA for the treatment of severe posterior epistaxis, with the goal of preserving collateral circulation and minimizing the risks associated with nonselective and/or bilateral ArtE. By analyzing clinical outcomes, recurrence rates, and procedural safety, this research seeks to clarify the role of unilateral superselective ArtE within the current treatment algorithm for posterior epistaxis and to highlight its potential as a first-line intervention in specific clinical scenarios.

## 2. Materials and Methods

### 2.1. Patient Selection

This prospective study was conducted in accordance with the Declaration of Helsinki, it was reviewed and approved by the Institutional Review Board (ORL-0122-3049), and written informed consent was obtained from all participants and the permission gained for the publication of their data.

This prospective study included patients who underwent superselective unilateral ArtE of the SPA for refractory posterior epistaxis between 2020 and 2024 at the Radiology Unit of Morgagni-Pierantoni Hospital, Forlì, Italy. The inclusion criteria comprised patients with severe posterior epistaxis that was unresponsive to at least two tamponade attempts or required hospital admission for bleeding control. Patients with anterior epistaxis or contraindications to ArtE (e.g., uncorrected coagulopathy, intracranial vascular anomalies) were excluded from the study. This study exclusively evaluated embolization, without direct comparison to other treatment modalities.

### 2.2. Angiographic Procedure

Under local anesthesia with mild sedation, vascular access was obtained via the right femoral artery using a 5-Fr introducer sheath. In cases where femoral access was not feasible, an alternative right radial artery approach was performed. A 5-Fr guiding catheter was advanced into the external carotid artery (ECA) to facilitate selective angiographic assessment. Following the catheterization of ECA, an initial angiographic study was conducted to evaluate the vascular anatomy and identify potential collateral circulation and target vessels contributing to epistaxis. This step was crucial for ensuring precise ArtE while minimizing the risk of non-target embolization to critical structures.

A microcatheter was advanced into the IMA to achieve the superselective catheterization of the SPA. Specifically, the microcatheter was positioned in the proximal segment of the SPA, which, in the anteroposterior projection, corresponded to the level of the sphenopalatine foramen. Arterial embolization was performed using 250–355 µm polyvinyl alcohol (PVA) particles under continuous fluoroscopic guidance. The endpoint of ArtE was defined as the complete occlusion of the SPA without reflux into the intracranial circulation. Furthermore, post-procedural angiography was performed to assess the embolization rates of the other distal IMA branches (descending palatine artery [DPA] and the infraorbital artery [IO]), categorized as follows:

- Complete embolization (cE): complete occlusion of the artery;
- Partial embolization (pE): reduced vessel perfusion with persistent opacification after contrast injection;
- No embolization (nE): no significant reduction in vessel perfusion.

The overall fluoroscopy time was recorded for each patient.

All angiographic procedures were performed by interventional radiologists with over seven years of experience in this field.

### 2.3. Outcome Measures

The primary outcome was clinical success, defined as the absence of recurrent bleeding within 24 h post-embolization.

The secondary outcomes included recurrence at one month as well as the incidence of minor and major complications.

### 2.4. Statistical Analysis

Continuous variables were expressed as means ± standard deviations (SD), while categorical variables were presented as frequencies and percentages. Given the descriptive nature of the study and the absence of a comparison between treatment groups, no inferential statistical tests were performed. Only descriptive statistics were used to evaluate the primary (clinical success) and secondary outcomes (recurrence and complication rates).

## 3. Results

### 3.1. Patient Characteristics

A total of 32 patients (24 males, 75%) with a mean age of 72 years (range: 51–89) underwent superselective unilateral SPA embolization. All patients had undergone an otorhinolaryngology evaluation, which confirmed severe idiopathic posterior epistaxis. The laterality of epistaxis was evenly distributed, with 16 cases (50%) on the left side and 16 cases (50%) on the right side. In two cases of bilateral epistaxis (6.5%), a predominant side of hemorrhage was identified (right predominance) using nasal endoscopy. All patients had initially received nasal tamponade as the first-line approach, and 16 out of 32 patients (50%) had also undergone previous endoscopic cauterization.

Among the risk factors, hypertension was the most prevalent, occurring in 22 out of 32 of cases (69%). The mean pre-procedural systolic blood pressure among these 22 patients was 160 mmHg (range: 130–200 mmHg). A total of 56% of patients (18/32) were receiving anticoagulant or antiplatelet therapy, with the following breakdown:

- 4/32 patients (12.5%) were on anticoagulation therapy;
- 10/32 patients (31%) were receiving antiplatelet therapy;
- 4/32 patients (12.5%) were on both treatments.

Twelve patients (38%) presented with both hypertension and anticoagulant/antiplatelet therapy as combined risk factors. Additionally, comorbidities recorded in the study population included diabetes mellitus in eight patients (25%), obstructive sleep apnea syndrome (OSAS) in five patients (15.5%), active smoking in nine patients (28%), and allergic rhinitis in eleven patients (34.5%).

Only three patients (9.5%) exhibited epistaxis without known risk factors.

Previous functional nasal surgery was reported in 4 out of 32 patients (12.5%). These procedures included septoplasty and turbinoplasty, performed to improve nasal airflow or to treat chronic nasal obstruction (Table 1).

### 3.2. Technical Success and Arterial Embolization Outcomes

Prior to the angiographic procedure, all patients underwent clinical stabilization, including the pharmacological management of elevated blood pressure according to current guidelines on the management of hypertension [13].

All patients underwent unilateral and superselective SPA embolization following the advancement of a microcatheter into the pterygopalatine segment of the IMA. Diagnostic angiography was performed in all cases.

No cases of active bleeding were documented; however, mucosal hyperemia was identified in 20 out of 32 patients (62%). No patients exhibited shunting or anastomoses with the intracranial circulation.

**Figure 1:**
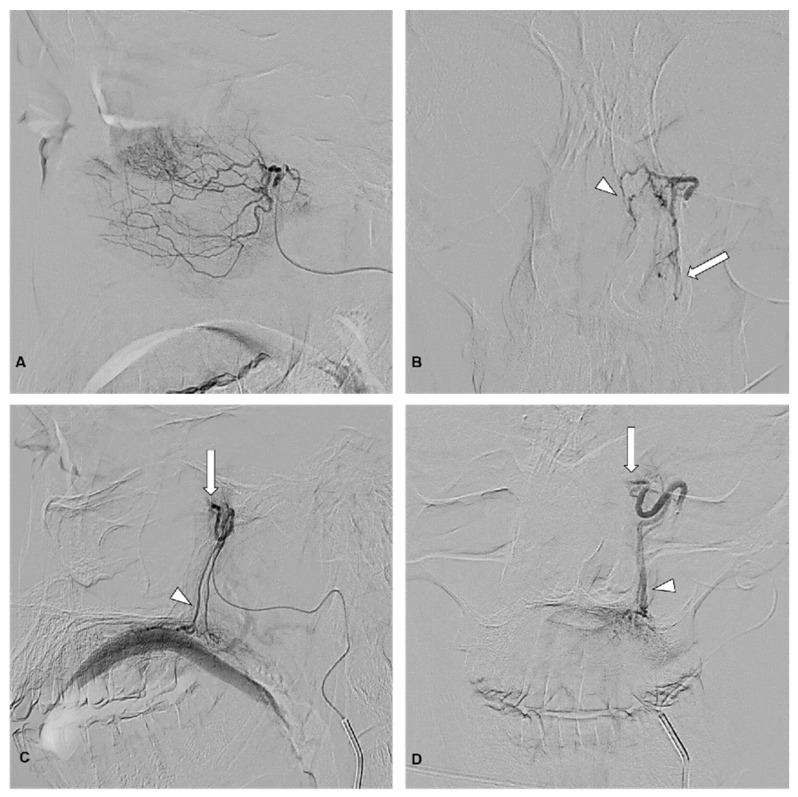
(**A**) Lateral and (**B**) anteroposterior angiographic views obtained following catheterization of the sphenopalatine artery (SPA). No active hemorrhage was observed. In (**B**), lateral branches supplying the turbinate (arrow) and medial branches supplying the nasal septum (arrowhead) are visible. Panels (**C**) (lateral view) and (**D**) (anteroposterior view) show post-embolization images after superselective SPA embolization with polyvinyl alcohol particles, demonstrating the complete occlusion of the SPA (arrow) with the preservation of the descending palatine artery patency (arrowhead).

**Figure 2:**
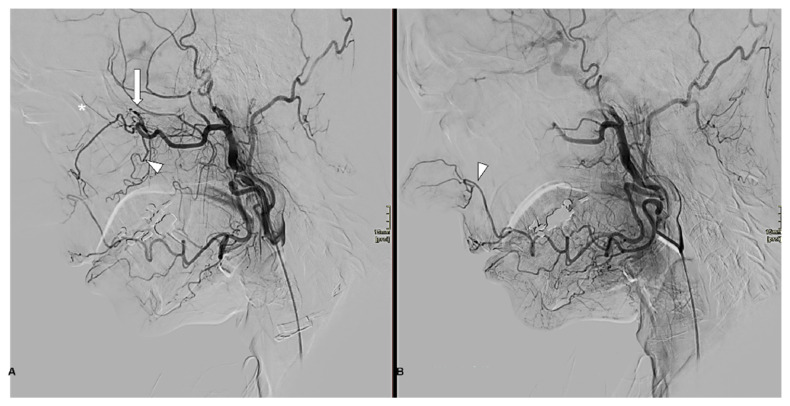
(**A**) Lateral angiographic projection obtained after the catheterization of the right external carotid artery. The image shows distal branches of the pterygopalatine segment of the internal maxillary artery: the sphenopalatine artery (SPA, arrow), the descending palatine artery (DPA, arrowhead), and the infraorbital artery (IO, asterisk). (**B**) Following superselective SPA embolization (arrow) with 250–355 μm polyvinyl alcohol particles, the complete occlusion of the SPA, DPA, and IO is observed, likely due to the proximal reflux of embolic material. Note that the right facial artery (arrowhead) provides vascular supply to the anterior portion of the nasal cavity.

Post-embolization imaging documented complete SPA occlusion in all 32 patients (100%) (Figure 1). Additionally, the following was found:

- cE of DPA was documented in 10 patients (31%), while partial embolization (pE) was observed in 12 patients (38%);
- cE of IO was noted in 10 patients (31%), with partial embolization (pE) in 2 patients (6%);
- Embolization of all three vessels was recorded in 10 patients (31%) (Figure 2).

Nasal tamponade was removed two hours post-procedure in all patients.

The mean procedural time was 19 ± 11 min.

### 3.3. Clinical Outcomes

Clinical success was achieved in 100% of cases. Two patients (6%) experienced a recurrent bleeding episode within seven days, necessitating hospital readmission and tamponade placement. However, neither case of rebleeding required major intervention, such as re-embolization. These two patients both had hypertension and were on dual antiplatelet and anticoagulant therapy.

Minor complications were reported in two patients (6%), both of whom presented with mild nasal dryness, which resolved within six months post-treatment. No major complications, such as visual disturbances, soft tissue necrosis, or severe facial pain, were observed.

The median follow-up period was 18 months (range: 6–38 months). No additional recurrences were reported beyond the single early recurrence and no long-term adverse effects attributable to ArtE were observed during follow-up.

## 4. Discussion

The results of this study support the efficacy and safety of superselective unilateral ArtE of the SPA for the management of severe posterior epistaxis. Our findings demonstrate a high rate of immediate hemostasis, with a 100% clinical success rate within the first 24 h post-procedure and a rebleeding rate of 6%. These results align with the previous literature, which has highlighted the benefits of interventional embolization in cases refractory to conservative and surgical management. A recent case series of 35 patients reported an immediate success rate of 97.5%. Additionally, a meta-analysis of 41 articles involving 1632 patients found that immediate success was achieved at a pooled mean of 90.9% (95% CI: 90.4–91.4), while rebleeding occurred at a pooled mean of 17% (95% CI: 16.5–17.5) [14].

Several studies have confirmed the correlation between blood thinners and an increased risk of failure following endoscopic ligation and endovascular embolization in the treatment of intractable epistaxis. In fact, due also to advanced age, patients referred for embolization represent a high-risk population, with an increased likelihood of treatment failure in at least 80% of cases [1,2,14,15,16]. In our series, the majority of patients were hypertensive (69%) or required chronic anticoagulation or antiplatelet therapy (56%). Additionally, 38% of our cohort had both of these two risk factors, increasing the likelihood of treatment failure or complications. However, despite these risk factors, unilateral ArtE achieved a low recurrence rate (6%) at one month.

Although the endovascular therapy of epistaxis is effective, there remains a potential risk of complications associated with this technique. The major complications reported in the literature include the following: necrosis of soft tissues (1.2%), stroke (1.1%), permanent blindness (0.4%), and facial nerve palsy (0.2%). Meanwhile, minor complications (6–45%) are largely transient and include the following: facial pain (13.1%), headaches (2.8%), paresthesia/numbness (1.4%), groin pain, and groin hematoma. Other rare complications are nasal septum perforation, sinusitis, and ear infection [1,10,11,17]. A cerebrovascular accident remains the most feared complication, with a higher risk in endovascular treatment (0.9%) compared to surgery (0.1%) [9]. This event is caused by unsuccessful embolization, which may result from the use of particles that are too small or which reflux into the intracranial circulation. The nasal fossa is a border vascular area between the intra- and extracranial circulation, necessitating a thorough understanding of vascular anatomy and close attention to flow dynamics. These dynamics can change in cases of internal carotid artery (ICA) atherosclerosis, vasospasm, or the rapid saturation of the proximal artery segment following stop-flow embolization.

Historically, embolization has been performed in a nonselective and bilateral manner involving multiple vascular territories, such as the IMA and the FA. In a clinical trial, Gottumukkala et al. [10] evaluated the association between the number of embolized vessels and the clinical outcome. The authors demonstrated that as the number of embolized ECA branches increased from one to four (ipsilateral IMA, bilateral IMA, bilateral IMA plus ipsilateral FA, or bilateral IMA plus bilateral FA), there was a linear decrease in the rate of early recurrent bleeding from 25% to 0%. However, this was accompanied by an increase in the rate of minor complications from 0% to 56% [10].

More recent studies have also reported on nonselective and/or unilateral and bilateral embolization techniques. Huyett P. et al. [11] treated 46 out of 54 patients (88.9%) with bilateral IMA embolization, while the remaining patients underwent unilateral IMA embolization. Additionally, 25 patients underwent FA embolization, with 6 receiving bilateral treatment and 18 receiving unilateral treatment. The major complication rate was 7.4%, including transient stroke, diplopia, and facial skin necrosis [11]. Similarly, Reyre et al. [8] reported a 10–15% incidence of complications following bilateral IMA embolization, which included transient facial pain and mucosal necrosis.

Therefore, based on these published series, bilateral embolization has been associated with an increased risk of ischemic complications, including mucosal necrosis and sensory disturbances.

In contrast, our study, which utilized a unilateral and superselective approach, demonstrated a markedly lower complication rate (6%), limited to minor nasal dryness, with no cases of significant ischemic events or neurologic sequelae. Furthermore, our findings are consistent with those of Brinjikji et al. [9], who analyzed trends in epistaxis embolization and reported favorable outcomes with selective embolization. The high clinical success rate observed in our cohort suggests that a unilateral approach may be sufficient in most cases, potentially reducing the need for bilateral intervention and lowering morbidity risks. Despite the superselective embolization of SPA, our study also documented non-target embolization due to reflux in the DPA in 69% and in the IO in 37% of cases. However, the cE or pE of other branches of the pterygopalatine segment of the IMA did not increase the risk of complications. In the future, the use of artificial intelligence in the clinical practice of radiology departments may enable the identification of posterior epistaxis laterality directly from imaging, thereby expediting the management of these patients [12].

In the literature, there is no consensus regarding the material to be used for embolization [9,10,18,19]. Some studies have employed different materials, including both liquid agents and coils [18,19,20]. However, the choice of embolic agent may significantly influence outcomes, especially in patients receiving anticoagulant therapy. For example, coils rely on the induction of thrombosis within the vessel to achieve occlusion, a mechanism that may be less effective in anticoagulated patients, leading to higher failure rates. Conversely, liquid embolic agents or particles occlude the vessel by obstruction, making them independent of the patient’s coagulation profile and potentially more effective in this subgroup.

Furthermore, a standardized aspect of our procedure is the consistent use of PVA, with particle sizes that are not excessively small, unlike other reports [10], to prevent migration through undetected and undetectable shunts. This standardized approach enhances the reproducibility of our procedure, provided that further studies confirm our findings.

Another important consideration regarding superselective unilateral ArtE is procedural time and radiation exposure. To our knowledge, these data have not been previously reported in the literature. Our mean fluoroscopy time of 19 min is comparable to or lower than that reported in other embolization studies [21], suggesting that experienced operators can perform this technique efficiently while minimizing the radiation exposure to both patients and clinicians.

Despite its strengths, our study has several limitations. First, our sample size, though adequate to demonstrate efficacy, remains relatively small compared to large-scale multicenter studies. Moreover, the study is single-center in design, which may limit the generalizability of the results to other settings or patient populations. A prospective, randomized comparison between unilateral and bilateral embolization would provide more robust data to determine the optimal embolization strategy. Another limitation is the lack of a long-term follow-up beyond 18 months. While no recurrences were observed after the initial 30-day period, further research is needed to assess the occurrence of late recurrences and to identify specific patient subgroups at higher risk. In addition, no post-treatment imaging was performed to evaluate nasal revascularization or the potential development of collateral circulation, which may influence long-term outcomes. Moreover, no post-treatment evaluation of blood pressure levels was conducted, despite hypertension being the most frequent risk factor identified in our cohort. These aspects will need to be addressed in future studies to better define the predictors of recurrence and treatment success.

In conclusion, superselective unilateral SPA embolization is a highly effective and safe intervention for severe posterior epistaxis, providing excellent immediate and short-term hemostasis with minimal complications. Given its advantages over nonselective or bilateral embolization and surgical alternatives, this approach should be considered a first-line interventional strategy, particularly for patients with high-risk factors, such as those on anticoagulant therapy or with recurrent bleeding despite endoscopic interventions. Future studies should focus on long-term outcomes and comparative analyses of alternative treatment modalities and interventional approaches to further refine management guidelines for posterior epistaxis. This prospective study demonstrates that unilateral superselective embolization of the SPA is a highly effective and safe treatment for severe posterior epistaxis, achieving a 100% immediate hemostasis rate and a low recurrence rate (6%), even in a high-risk population characterized by hypertension and antithrombotic therapy. Furthermore, the procedure was associated with minimal complications, with no major ischemic or neurological adverse events reported.

The findings support the adoption of a targeted, vessel-sparing endovascular approach, which may reduce the need for bilateral or nonselective embolization strategies historically associated with higher morbidity.

Given the limitations of a single-center design and the lack of post-treatment imaging or a long-term follow-up beyond 18 months, future multicenter, randomized studies are warranted. These should explore long-term efficacy, compare embolization strategies (unilateral vs. bilateral, selective vs. non-selective), and assess the role of imaging and clinical predictors in optimizing treatment selection and outcomes in posterior epistaxis.

## Figures and Tables

**Table 1:** Patient characteristics.

Characteristic	N (%)
Mean age (years)	72 (mean)
Male sex	24 (75%)
Hypertension	22 (69%)
Mean systolic blood pressure (mmHg)	160 (range: 130–200)
Anticoagulant therapy	4 (12.5%)
Antiplatelet therapy	10 (31%)
Dual antithrombotic therapy	4 (12.5%)
Hypertension + antithrombotics	12 (37.5%)
Diabetes mellitus	8 (25%)
Obstructive sleep apnea syndrome (OSAS)	5 (15.5%)
Active smoking	9 (28%)
Allergic rhinitis	11 (34.5%)
No identifiable risk factors	3 (9.5%)
Previous nasal surgery	4 (12.5%)
Epistaxis side (left)	16 (50%)
Epistaxis side (right)	16 (50%)
Bilateral epistaxis with unilateral predominance	2 (6.5%)
Nasal packing before embolization	32 (100%)
Previous endoscopic cauterization	16 (50%)

## Data Availability

The original contributions presented in this study are included in the article. Further inquiries can be directed to the corresponding author.
